# Fibular Nonunion: A Systematic Review of Incidence, Diagnosis, and Treatment Outcomes

**DOI:** 10.3390/jpm16070373

**Published:** 2026-07-10

**Authors:** Virginia Cinelli, Federico Moretti, Chiara Comisi, Antonio Mascio, Gloria Assegbede, Vincenzo La Vergata, Giulio Maccauro, Carlo Perisano, Tommaso Greco

**Affiliations:** 1 Department of Orthopedics and Geriatric Sciences, Catholic University of the Sacred Heart, Largo Francesco Vito, 8, 00168 Rome, Italycarlo.perisano@policlinicogemelli.it (C.P.); 2 Department of Orthopedics, Ageing and Rheumatological Sciences, Fondazione Policlinico Universitario A. Gemelli IRCCS, Largo Agostino Gemelli 8, 00168 Rome, Italy; 3 Department of Life Sciences, Health, and Healthcare Professions, Link Campus University, 00165 Rome, Italy

**Keywords:** fibula, fracture healing, treatment outcome, nonunion, pseudoarthrosis, reoperation

## Abstract

**Background:** Fibular nonunion is an uncommon but clinically relevant complication following fractures or surgical procedures, often resulting in persistent pain and functional impairment. Due to its rarity, current evidence remains limited and no standardized treatment guidelines are available. **Purpose:** To systematically review the literature on fibular nonunion, focusing on clinical presentation, diagnostic approaches, and treatment outcomes. **Methods:** A systematic review was conducted in accordance with PRISMA guidelines. MEDLINE, Scopus, and Web of Science were searched up to July 2025. Studies including adult patients (≥18 years) with fibular nonunion treated either conservatively or surgically were included. Data regarding demographics, clinical presentation, and treatment outcomes were extracted and analyzed descriptively. **Results:** Nineteen studies comprising 183 patients were included. The mean patient age was 45.7 years, with a predominance of males (58.4%). The distal third of the fibula was the most frequently involved site (75.9%). The mean time to diagnosis was 28.6 weeks. Surgical treatment was performed in 65.6% of cases, most commonly using open reduction and internal fixation. Among studies reporting union outcomes, favorable radiographic healing rates were observed following surgical treatment. Conservative treatment was primarily reserved for asymptomatic or minimally symptomatic patients. The overall complication rate was low (3.8%), mainly consisting of minor infections and hardware-related issues. **Conclusions:** Fibular nonunion is an uncommon but clinically significant condition. Available evidence suggests that surgical management may represent the most consistently successful treatment strategy in symptomatic and mechanically unstable cases, while nonoperative treatment may remain appropriate in carefully selected asymptomatic or minimally symptomatic patients. However, the available literature is limited by retrospective study designs, heterogeneous populations, inconsistent outcome reporting, and variable definitions of nonunion, highlighting the need for prospective multicenter studies and standardized treatment protocols.

## 1. Introduction

Fibular nonunion is a relatively rare but clinically significant complication that can occur following fractures or surgical interventions involving the fibula [1].

Fibular fractures have an annual incidence of approximately 74 cases per 100,000 people, occurring predominantly in women over the age of 40, particularly in the postmenopausal population [2]. According to the current literature, the rate of fibular nonunion ranges between 0.2% and 5.4%, depending on the fracture type, treatment method, and the presence of associated injuries [1].

Regarding elective surgical procedures, the fibula may be involved in association with tibial osteotomies for the correction of knee and ankle varus or valgus deformities, for limb lengthening, or during bone transport using circular external fixator (EF) [3]. The incidence rate of fibular nonunion after osteotomy varies according to the surgical technique and the clinical context. In fibular osteotomy performed as part of a closed-wedge high tibial osteotomy, the literature reports an incidence of approximately 13.9%, with about 10% of cases requiring surgical revision [4]. In the setting of distraction osteogenesis, fibular nonunion occurs in roughly 15.5% of cases, with a higher risk observed in situations involving greater lengthening or concomitant tibial nonunion [5].

Fibular nonunion may lead to persistent pain, functional impairment, ankle instability, and reduced quality of life. Early diagnosis and appropriate management are essential to prevent deformities, provide pain relief, and restore limb functionality.

The pathophysiology of nonunion is relatively well understood. Bone healing is closely linked to adequate vascularization, mechanical stability, and the biological environment at the fracture site [6,7]. Additionally, known factors such as smoking, obesity, infections, diabetes mellitus, alcohol use [8], and advanced age can impact the bone healing process [9,10]. In most cases, standard radiographic examination is sufficient for the diagnosis of nonunion; however, in some instances, a CT scan is required for a more accurate assessment of the bone healing status [11].

Being an uncommon complication, fibular nonunion is poorly represented in the literature, and available data remains limited. There is currently no well-defined guideline for the treatment of fibular nonunion. Conservative instrumental therapies such as magnetotherapy are often attempted [12] but surgical treatment is much more frequently required to resolve pain symptoms and improve ankle stability [13].

The aim of this systematic review was to evaluate the existing evidence related to fibular nonunion. We examined the incidence, demographics data, assessment methods, and treatment options documented in the current literature.

## 2. Materials and Methods

This review was conducted in accordance with the Preferred Reporting Items for Systematic Reviews and Meta-Analyses (PRISMA) guidelines [14], ensuring a comprehensive and systematic approach to data collection and analysis. The review protocol was prospectively registered in the International Prospective Register of Systematic Reviews PROSPERO (CRD420251116343) before study initiation.

The literature search was conducted across three online databases: PubMed, Scopus, and Web of Science. The keywords used for the research were combined as follows: (“fibula” OR “fibular”) AND (“nonunion” OR “non-union” OR “pseudarthrosis” OR “delayed union”) AND (“treatment”).

All retrieved articles, titles, and abstracts were carefully screened to determine their eligibility for inclusion. All studies involving adult human subjects, published in English, with publication dates up to July 2025 were collected.

In cases of uncertainty, the full texts of articles were obtained for further assessment. The senior author, along with subject matter experts, reviewed all full-text articles to minimize potential bias arising from preconceptions. To ensure thoroughness, the reference lists of pertinent studies were also examined to identify additional relevant publications.

Two reviewers (V.C. and C.C.) independently screened the abstracts and retrieved full texts for any inconclusive abstracts. Any discrepancies between their assessments were resolved through discussion, and if consensus could not be reached, the senior authors (C.P or T.G.) were consulted. The references of the selected articles were manually checked to locate further relevant studies. All chosen articles were then analyzed retrospectively by three authors (F.M., A.M., and V.C.), who extracted and documented the data in an Excel spreadsheet. This dataset was subsequently reviewed by four authors (V.C., C.C., F.M., A.M.) to ensure consistency and agreement on the extracted information. Additionally, references from the identified articles were searched for further relevant studies, considering all relevant journals in the field.

### 2.1. Inclusion and Exclusion Criteria

The criteria established for selecting studies aimed to ensure the inclusion of research with rigorous methodology and comprehensive reporting standards. We targeted studies involving adult patients aged 18 years or older who were treated for fibular nonunion. Accepted study designs included retrospective and prospective case series, controlled clinical trials, as well as both quasi-randomized and randomized controlled trials. Only articles published in English that detailed clinical, radiological, or complication outcomes with at least 12 months of follow-up were eligible. Additionally, all studies needed to be accessible through institutional or publicly available journal databases.

Case reports, expert opinions, previous systematic reviews, letters to the editor, similar meta-analyses, and studies with incomplete data were excluded from the study because unsuitable for data pooling.

No universally standardized definition of fibular nonunion was consistently applied across all included studies. When explicitly reported, the diagnosis was generally based on persistent clinical symptoms associated with absent radiographic progression of healing. In studies where no formal definition was provided, fibular nonunion was interpreted according to commonly accepted orthopedic criteria, typically defined as failure of fracture healing within approximately 9 months, with no radiographic progression over at least 3 consecutive months.

### 2.2. Study Assessment

The methodological quality of each study was evaluated using the Methodological Index for Non-Randomized Studies (MINORS) [15], which has a maximum score of 24 points for comparative studies and 16 points for non-comparative studies. Two authors (C.C and F.M.) independently assessed the studies and resolved any discrepancies through consensus to determine the final MINORS score.

### 2.3. Statistical Analysis

From each study the following data were collected: number of patients with nonunion, incidence, gender, age, site of fibular nonunion, type of previous treatment, symptoms, diagnostic assessments, time to diagnosis and clinical outcomes.

Statistical analyses were performed using IBM SPSS Statistics (version 31.0.1.0; IBM Corp., Armonk, NY, USA). Basic descriptive calculations were carried out, including sums, arithmetic means, and weighted means. The results were reported as mean values, rounded to one decimal place, to provide a concise and accurate description of the collected data.

Parameters such as incidence, patient age, and time to diagnosis were calculated using a weighted mean, allowing the results from individual studies to be combined while accounting for differences in sample size. This approach provides a more representative estimate of the overall population.

## 3. Results

### 3.1. Search and Selection Process

**Figure 1:**
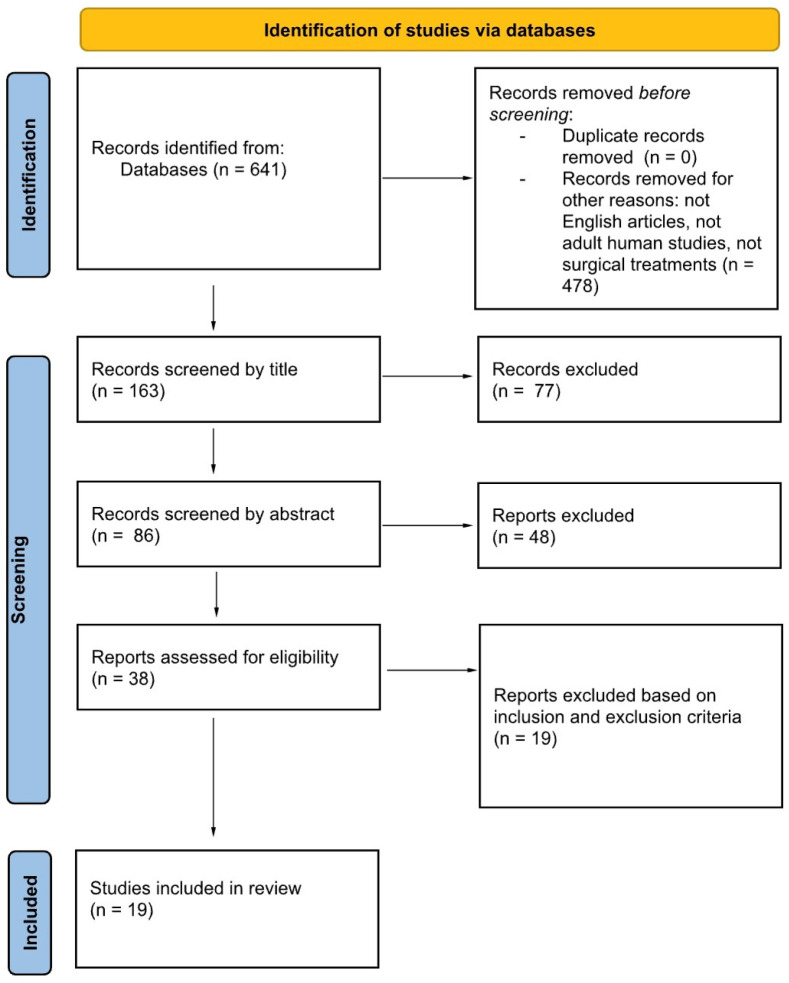
PRISMA flowchart.

The study flowchart is presented in Figure 1. We found 641 articles in the initial literature search and no duplicated studies. Among these, 163 were deemed relevant based on title. The studies were then screened by abstract, after which 86 articles were selected for full-text review. Finally, following a complete reading and application of inclusion and exclusion criteria, 19 articles were considered eligible and included in the systematic review. The mean MINORS score calculated for the included studies was 7.8. Most studies lost MINORS points because of retrospective design, lack of prospective data collection, absence of unbiased outcome assessment, and incomplete follow-up reporting.

### 3.2. Demographics Data

Out of the 19 articles included in the study, a total of 183 cases of fibular nonunion were identified, including 25 occurring after corrective osteotomy. The mean incidence of nonunion among patients in the sample with fractures was 2.85%, whereas among patients who underwent osteotomy, the incidence of nonunion was significantly higher, at 14.5%. The mean patient age was 45.7 years, and males accounted for most cases (58.4%).

Nonunion was symptomatic in 82.5% of patients, most commonly presenting with pain.

Diagnosis was established in all cases based on clinical examination, X-rays were obtained in 100% of cases and were sufficient to confirm nonunion in 79.8%. In the remaining cases, CT scan was performed to complete the diagnostic work-up.

The diagnosis of nonunion was made at a mean of 28.6 weeks post-injury or post-procedure, during which patients typically underwent protected weight-bearing restrictions, immobilization, and physiotherapy without symptomatic improvement.

The site of the nonunion was distal in 75.9% of cases, mid-shaft in 7.1%, and proximal in 8.7% (with location not specified in 8.1% of cases) (Table 1).

To better account for the heterogeneity of the included population, patients were also analyzed according to the etiology of fibular nonunion (fracture-related versus osteotomy-related). Fracture-related nonunion accounted for 158 cases, whereas 25 cases occurred following corrective osteotomies. Osteotomy-related nonunion showed a higher reported incidence (14.5% vs. 2.85%) and a longer mean time to diagnosis (39.4 vs. 30.3 weeks). Differences were also observed in symptom prevalence and treatment distribution between the two groups (Table 2).

Several studies incompletely reported demographic and clinical variables, including age, sex, previous treatment, and time to diagnosis (Supplementary File). Consequently, weighted mean calculations should be interpreted cautiously, as missing data may have influenced pooled estimates.

### 3.3. Type of Treatment and Outcomes

Of the 183 patients, 120 (65.6%) underwent surgical management and other 63 (34.4%) underwent conservative treatment (Table 3).

The most common procedure was open reduction and internal fixation (ORIF), with or without bone grafting, either autologous or allogenic performed in 97 patients (53%). About ORIF, it was performed in 4 patients using an intramedullary nail, in 12 patients with percutaneous screws, in 4 patients with a lag screws, while a plate was used in the remaining 77 patients (Table 4).

Other surgical treatments performed in the study included the following: six patients were treated with EF (3.2%), six patients (3.2%) treated exclusively with bone graft (BG), two patients (1.0%) who underwent ankle joint arthrodesis, one patient (0.5%) who underwent excision, one patient (0.5%) treated with drilling, and finally seven patients (3.8%) who received a segmental resection.

In the included studies, excision generally referred to removal of a limited symptomatic nonunion fragment, whereas segmental resection involved excision of a larger fibular segment (partial fibulectomy).

Among studies reporting radiographic union, surgical treatment demonstrated consistently high healing rates, frequently accompanied by symptoms improvement.

Among the 63 patients managed nonoperatively, treatment most consisted of observation or watchful waiting, particularly in asymptomatic or minimally symptomatic individuals. However, reporting of conservative management outcomes was inconsistent across studies. Several authors provided only limited information regarding symptom progression, radiographic evolution, or subsequent conversion to surgery. Consequently, the effectiveness of nonoperative treatment could not be reliably quantified, and conclusions regarding conservative management should be interpreted cautiously.

### 3.4. Complications

Complications were observed in seven patients (3.8%), all of whom sustained a fracture of the distal one-third of the fibula.

## 4. Discussion

Bone nonunion is defined as the failure of a healing process following osteotomy and particularly fracture, within the expected time frame, typically considered to be 9 months from the injury with no evidence of progressive healing for at least 3 consecutive months [31]. Fibular nonunion is an uncommon but clinically significant complication, leading to chronic pain, local tenderness, instability, gait impairment, and residual deformity [32]. Among the several surgical complications, nonunion occurs rarely, also according to fracture type, location, initial management, fixation method, and associated injuries [4,33]. Although the fibula is a non-weight-bearing bone, its integrity is essential for ankle stability and load distribution [34]. The etiology of nonunion is multifactorial and includes mechanical factors (such as insufficient fixation or persistent micromotion), biological factors (involving poor vascularization and inadequate bone contact), as well as patient-related factors such as smoking, diabetes, comorbidities, local tissue conditions, and infections [13].

The predominance of distal fibular nonunion observed in this review may be explained by several anatomical and biomechanical factors. Distal fibular fractures are frequently associated with rotational ankle trauma, syndesmotic instability, and repetitive torsional stress during gait [35]. Furthermore, the distal fibula has limited soft tissue coverage and vascular vulnerability following trauma or surgery, potentially impairing biological healing and increasing the risk of persistent micromotion [36].

The prevention of nonunion relies on fundamental principles such as adequate preparation of the bony surfaces, proper alignment, and optimal mechanical stability, which have been shown to be crucial in achieving high fusion rates in arthrodesis procedures [37].

Being an uncommon complication, fibular nonunion is poorly represented in the literature, and available data remains limited. The purpose of this systematic review was to analyze the current evidence on fibular nonunion, with particular attention to the different treatment strategies adopted according to the type of fracture and the initial management.

An important consideration is the marked heterogeneity of the included population. Fibular nonunion occurring after isolated ankle fractures, tibial shaft fractures, and corrective osteotomies likely represents biologically and biomechanically distinct clinical entities. Nevertheless, given the rarity of fibular nonunion and the limited number of available studies, all clinically relevant etiologies were included to provide the most comprehensive overview currently possible. This heterogeneity inevitably limits the direct comparability of outcomes and should be considered when interpreting pooled results.

Due to the few data about it, we cannot establish which is the majority cause that leads to a nonunion, but instead we can do several considerations. Firstly, consolidation failure in a conservative setting is often attributable to inadequate mechanical stability at the fracture site. Although isolated fibular fractures are considered less critical for axial loading than the tibia (since the fibula supports a minor portion of the body weight), rotational stability and optimal apposition of the bone ends remain essential prerequisites for healing [18]. Elliot et al. in their study propose a “bone-healing nonunion theory”, describing nonunion as a failure of the functional biological system that develops around a fracture site to respond adequately to its mechanical and biological environment [38]. According to this concept, nonunion are not merely cases of “absent healing” but rather represent an inability of the bone-healing unit to progress through its normal reparative stages due to dysfunction in one or more of its key components, such as mechanical, biological, or vascular. Consequently, conservative treatment may fail to provide adequate mechanical stability. Other studies have reported fibular nonunion occurring after surgically managed fractures or as iatrogenic complications following corrective osteototomies [6,9]. Also in this case, several contributing factors have been identified, including residual mechanical instability, inadequate or insufficient osteosynthesis, loss of reduction, and impaired biological healing, often compounded by intrinsic patient-related factors.

Regarding the surgical management of fibula nonunion, it remains a particularly challenging topic due to the lack of robust evidence and high-quality literature. Most available data derive from isolated case reports and from small retrospective series that are often heterogeneous in terms of etiology, anatomical location, patient age, previous surgeries, and follow-up duration [39]. This limited and fragmented literature represents both the main limitation of our review and one of its strengths, as, to the best of our knowledge, this is among the very few systematic reviews available on this topic. The only previous review identified was conducted by Bhadra et al. [1] in 2012, who included twelve studies and reported an incidence of fibular nonunion ranging from 0.3% to 5.4%, with similar risk factors and etiologies to those identified in our analysis. Most cases occurred in the distal third of the fibula and were frequently associated with tibial shaft fractures treated with intramedullary nailing, as confirmed also in our analysis. Treatment strategies varied according to symptom severity and fracture characteristics, ranging from conservative observation to surgical approach, with or without bone grafting [4].

Our results suggest favorable outcomes following surgical treatment, accounting for approximately 65.6% of cases managed surgically compared with conservative approaches. The apparent superiority of surgical treatment should also be interpreted cautiously because of potential selection bias. Symptomatic patients with instability, persistent pain, deformity, or functional limitation were inherently more likely to undergo operative treatment, whereas asymptomatic or minimally symptomatic patients were preferentially managed conservatively. Consequently, direct comparison between operative and nonoperative outcomes remains limited.

For instance, Batten et al. reported 12 cases of fibular nonunion, treated with percutaneous screws [18]. Their study demonstrated excellent outcomes, with only one superficial wound infection and one intraoperative fracture and a final union rate of 100%. Similarly, Donken et al. reported eight cases of fibular nonunion following a Weber B fracture, of which only one was managed conservatively [21]. The remaining seven were treated surgically using various techniques, including lag screws, plating, and bone grafting, achieving successful union in all cases. According to the authors, one of the major risk factors for nonunion is hidden instability, and consequently misdiagnosis that predispose to a mechanical failure and healing failure.

In cases of distal fibular nonunion, most reported patients treated with plate fixation combined with autologous bone grafting achieved radiographic union, frequently accompanied by clinical improvement when reported.

Another important limitation concerns outcome assessment. Most studies primarily reported radiographic union, while standardized functional scores and patient-reported outcome measures were inconsistently evaluated. Therefore, successful radiographic healing may not necessarily correlate with complete symptom resolution or restoration of function.

Autologous bone grafting may be considered in cases characterized by bone loss, atrophic nonunion patterns, or previous failed fixation. Across the included studies, available studies suggest favorable radiographic healing rates following stable fixation combined with biological augmentation and improved functional outcomes compared with conservative or minimally invasive techniques, especially in symptomatic and mechanically unstable cases.

The most reported adverse events include superficial wound infections, hardware irritation, and, less frequently persistent nonunion, loss of reduction, and incomplete patient satisfaction [13]. Batten et al. reported their experience with percutaneous screw fixation for the management of fibular nonunion [18]. Among 12 treated patients, one case of superficial wound infection and one intraoperative iatrogenic fracture were observed.

Similarly, Khurana et al. documented two superficial wound infections in a cohort of 12 patients treated with ORIF [9]. Konig et al. treated one of six patients using an intramedullary fibular nail; this patient subsequently developed a deep implant-related infection, requiring nail removal followed by EF until union was achieved [24]. External fixation was infrequently reported in the included studies and was primarily used in selected complex cases, including management of infection and mechanically challenging nonunion.

Sneppen et al. reported an increased risk of post-treatment ankle osteoarthritis, characterized by persistent pain and functional limitation, despite successful correction of the nonunion [30]. Conversely, Walsh et al. observed persistent pain in one patient who declined operative intervention and elected to pursue conservative management [13].

Recent studies have explored intramedullary fibular nailing as a minimally invasive alternative to traditional plating. The technique minimizes soft-tissue dissection, preserves periosteal blood supply, and potentially lowers the risk of wound complications. In the present review, Kavanagh et al. reported successful union in three patients treated with intramedullary nailing [23]. Similarly, isolated reports outside the present review have described satisfactory radiographic healing following fibular nailing, although implant-related irritation requiring hardware removal has also been reported [40]. Early clinical reports therefore suggest encouraging outcomes, particularly in elderly patients or in cases with compromised soft tissues [41,42,43,44]. However, the current evidence base remains limited to small case series and isolated case reports, and further comparative studies are required to establish its definitive role relative to conventional plating techniques. At present, fibular nailing should be considered a selective option in cases requiring minimal soft-tissue disruption or as a revision strategy.

In the present review, 63 of 183 patients (34.4%) were treated nonoperatively, through clinical and radiographic surveillance combined with symptomatic management. This approach was mainly reserved for asymptomatic or minimally symptomatic nonunion where fibular discontinuity did not compromise ankle function or alignment. Most patients maintained stable symptoms over time, without progression or surgical conversion. The use of low-intensity pulsed ultrasound or pulsed electromagnetic field therapy represents first-line, noninvasive therapeutic options that can be employed to promote bone healing and stimulate biological activity at the nonunion site [45,46]. However, persistent pain or mechanical instability predicted poorer outcomes, emphasizing that nonoperative management is reasonable only in selected, stable cases, provided that close follow-up is ensured.

However, more recent reports have introduced updated fixation techniques, minimally invasive approaches, and the adjunctive use of biological agents, which reflect ongoing evolution in the treatment paradigm. The findings of our review therefore confirm the crucial principles already outlined in the earlier literature while integrating recent advancements and highlighting persisting gaps. The limited number of contemporary studies underscores the need for multicenter collaborations and standardized reporting to better define optimal treatment strategies for this uncommon but clinically significant condition.

### 4.1. Limitations

This review has several limitations. The inclusion of studies published between 1965 and 2025 introduces substantial temporal heterogeneity. Over this period, fixation techniques, implant design, biological augmentation strategies, and rehabilitation protocols have evolved considerably. Consequently, older studies may not fully reflect contemporary surgical practice, limiting the generalizability of pooled estimates. The included studies were predominantly retrospective case series with relatively small sample sizes and heterogeneous patient populations. The overall methodological quality, as reflected by the mean MINORS score, was low to moderate.

Several demographic and clinical variables were incompletely reported across the included studies. Although weighted means were calculated using all available data, the substantial proportion of missing information for some variables may have influenced pooled estimates and should be considered when interpreting the results.

An additional limitation concerns the lack of a universally accepted definition of fibular nonunion. Although the FDA definition traditionally considers nonunion as the absence of healing progression within 9 months after injury, several studies included in this review diagnosed fibular nonunion substantially earlier, often between 14 and 20 weeks, based on persistent symptoms and radiographic findings. This variability likely contributed to heterogeneity across studies and limits direct comparison of outcomes. Future research would benefit from the adoption of standardized diagnostic criteria to improve comparability and facilitate the development of evidence-based treatment recommendations.

Additionally, variability in treatment approaches, follow-up duration, and outcome reporting limits the ability to draw definitive conclusions or perform meta-analysis.

An important limitation of the available literature is the scarcity of functional outcome reporting. Most studies focused primarily on radiographic union, whereas objective measures of ankle stability, range of motion, return to activity, patient-reported outcome measures (PROMs), and quality-of-life assessments were inconsistently reported or entirely absent. Given the biomechanical role of the fibula in ankle stability, functional outcomes may be more clinically relevant than radiographic healing alone. Consequently, successful radiographic union should not necessarily be interpreted as equivalent to complete functional recovery.

Publication bias must also be considered, as small retrospective case series with positive surgical outcomes are more likely to be reported than unsuccessful or conservatively managed cases.

### 4.2. Clinical Implications

From a clinical perspective, the findings of this review suggest a predominantly surgery-oriented treatment approach for symptomatic fibular nonunion. Patients presenting with persistent pain, mechanical instability, impaired ankle function, deformity, or failed conservative management appear to benefit most from operative treatment. In these cases, stable fixation—most achieved with plate osteosynthesis—with biological augmentation when necessary, was associated with favorable radiographic healing rates and symptom improvement when reported.

Conversely, nonoperative management may remain appropriate in carefully selected asymptomatic or minimally symptomatic patients with preserved ankle stability. However, close clinical and radiographic follow-up is essential, as persistent pain or functional deterioration may indicate underlying instability and the need for surgical intervention.

Based on the currently available evidence, treatment decisions should rely not only on radiographic findings but also on symptom severity, ankle stability, patient functional demands, and associated lower-limb pathology.

## 5. Conclusions

Fibular nonunion is an uncommon but clinically relevant condition associated with persistent pain, ankle instability, and functional limitation. Its etiology is multifactorial, involving both mechanical and biological factors.

Current evidence suggests favorable outcomes following surgical treatment in symptomatic or mechanically unstable fibular nonunion, particularly when stable fixation is combined with biological augmentation where necessary. Minimally invasive techniques, including percutaneous screw fixation and intramedullary fibular nailing, may represent promising alternatives in selected cases; however, evidence supporting their superiority over conventional plating remains limited.

Nonoperative treatment may still be appropriate in selected asymptomatic or stable nonunion, provided that careful clinical and radiographic monitoring is maintained.

Nevertheless, the currently available literature remains limited by retrospective designs, heterogeneous populations, inconsistent outcome reporting, and substantial methodological variability. Further prospective multicenter studies with standardized definitions and outcome measures are required to better define optimal treatment strategies for fibular nonunion.

## Figures and Tables

**Table 1:** Overview of demographics data. ORIF (open reduction internal fixation); CT (computed tomography); Fx (fracture); HTO (high tibial osteotomy); NR (not reported); * Symptoms primarily referred to pain and/or local tenderness.

	N Fibular Nonunion	Incidence	Type of Previous Treatment	Mean Age (Years)	Male	Diagnostic Assessment	Time to Diagnosis (Weeks)	Site of Fibular Nonunion	Symptoms *	MINORS Score
Ahmed (2007) [16]	3	NR	3 Conservative Fx (100%)	39	33.3%	3 X-rays 3 CT	18	3 distal ⅓	3 (100%)	8/16
Amos (2022) [17]	5	NR	5 conservative Fx (100%)	NR	20%	5 X-rays	37	5 distal ⅓	5 (100%)	8/16
Batten (2018) [18]	12	NR	12 conservative Fx (100%)	47	58.3%	12 X-rays	61	12 distal ⅓	12 (100%)	8/16
Böstman (1991) [19]	8	5.4%	8 conservative Fx (100%)	41.4	75%	8 X-rays	16	2 distal ⅓ 6 middle ⅓	5 (62.5%)	8/16
Brinker (2010) [20]	4	NR	1 fibular osteotomy (25%) 3 conservative Fx (75%)	47	50%	4 X-rays 4 CT	NR	2 distal ⅓ 2 middle ⅓	4 (100%)	7/16
Donken (2011) [21]	8	2.1%	8 Conservative Fx (100%)	49	37.5%	8 X-rays 4 CT	20	8 distal ⅓	8 (100%)	7/16
Ebraheim (1993) [22]	17	NR	NR	NR	88.2%	17 X-rays 4 CT	24	11 distal ⅓ 5 middle ⅓ 1 proximal ⅓	16 (94.1%)	7/16
Kavanagh (2025) [23]	3	NR	3 conservative Fx (100%)	47.3	100%	3 X-rays 3 CT	30	3 distal ⅓	3 (100%)	8/16
Khurana (2013) [9]	12	4.8%	8 ORIF (66.6%) 4 conservative Fx (33.3%)	43	33.3%	12 X-rays 12 CT	NR	12 distal ⅓	12 (100%)	9/16
König (1989) [24]	6	NR	NR	NR	NR	6 X-rays	NR	6 distal ⅓	6 (100%)	7/16
Jennison (2018) [5]	9	15.5%	9 osteotomy in distraction osteogenesis of the tibia (100%)	35.4	55.5%	9 X-rays	23.4	9 distal ⅓	3 (33.3%)	9/16
McGonagle (2010) [25]	3	NR	3 Conservative Fx (100%)	41.3	100%	3 X-rays 3 CT	14	3 distal ⅓	2 (66.6%)	7/16
Mendelsohn (1965) [26]	2	1.1%	NR	NR	NR	2 X-rays	NR	2 distal ⅓	2 (100%)	7/16
Ramanoudjame (2012) [4]	15	13.90%	15 HTO (100%)	54.3	NR	15 X-rays	49	15 proximal ⅓	11 (73.3%)	9/16
Shen (1993) [27]	15	4.5%	15 Conservative Fx (100%)	NR	NR	15 X-rays	NR	NR	4 (26.7%)	7/16
Siliski (1993) [28]	26	NR	NR	NR	NR	26 X-rays	NR	26 distal ⅓	26 (100%)	7/16
Sneppen (1971) [29]	23	0.3%	NR	45	91.3%	23 X-rays	NR	23 distal ⅓	17 (73.9%)	7/16
Sneppen (1971) [30]	6	NR	NR	45	NR	6 X-rays	28	6 distal ⅓	6 (100%)	9/16
Walsh (2004) [13]	6	NR	6 conservative Fx (100%)	51.5	16.6%	6 X-rays 4 CT	30.7	6 distal ⅓	6 (100%)	9/16
Total	183 (100%)	2.85% (fracture) 14.5% (osteotomy)	70 Conservative Fx (38.2%) 9 ORIF (4.9%) 25 osteotomy (13.6%) 79 N/A (43.1%)	45.7	58.4%	183 X-rays (100%) 37 CT (20.2%)	28.6	139 distal ⅓ (75.9%) 13 middle ⅓ (7.1%) 16 proximal ⅓ (8.7%) 15 NR (8.1%)	151 (82.5%)	7.8/16

**Table 2:** Subgroup Analysis. Fx (fracture); HTO (high tibial osteotomy); ORIF (open reduction internal fixation); BG (bone graft).

	Fx Nonunion	Post HTO Nonunion
N fibular nonunion	158	25
Mean age	45.3	47.2
Mean diagnosis time (weeks)	30.3	39.4
Symptoms	86.1%	60.0%
Conservative treatment	52 (32.9%)	11 (44.0%)
Surgical treatment	106 (67.1%): 84 ORIF, 6 EF, 6 BG only, 6 segmental resection 2 arthodesis, 1 excision, 1 drilling	14 (56.0%): 13 ORIF, 1 segmental resection

**Table 3:** Overview of treatments. EF (External Fixator); ORIF (Open reduction and internal fixation); BG (bone graft); NR (not reported).

	N Fibular Nonunion	Conservative Treatment	EF	ORIF ± BG	BG Only	Arthrodesis	Excision	Drilling	Segmental Resection	Complications	Union Rate (%)
Ahmed (2007) [16]	3	0	0	3	0	0	0	0	0	0	100%
Amos (2022) [17]	5	0	0	5	0	0	0	0	0	0	100%
Batten (2018) [18]	12	0	0	12 (percutaneous screw)	0	0	0	0	NR	1 wounds infection 1 intraop fracture	100%
Böstman (1991) [19]	8	7	0	0	0	0	0	0	1	0	100%
Brinker (2010) [20]	4	0	0	0	0	0	0	0	4	0	100%
Donken (2011) [21]	8	1	0	4 (lag screw) 2 (only plate) 1 (ORIF + BG)	0	0	0	0	0	0	0
Ebraheim (1993) [22]	17	2	4	8	3	0	0	0	0	0	100%
Kavanagh (2025) [23]	3	0	0	3 (nail)	0	0	0	0	0	0	100%
Khurana (2013) [9]	12	0	0	12	0	0	0	0	0	2 wounds infections	100%
König (1989) [24]	6	0	2	3 (plate) 1 (nail)	0	0	0	0	0	1 nail infection -> FE	NR
Jennison (2018) [5]	9	7	0	2	0	0	0	0	0	0	100%
McGonagle (2010) [25]	3	1	0	2	0	0	0	0	0	0	100%
Mendelsohn (1965) [26]	2	0	0	0	1	1	0	0	0	0	0
Ramanoudjame (2012) [4]	15	4	0	11	0	0	0	0	0	0	100%
Shen (1993) [27]	15	13	0	0	0	0	0	0	2	0	0
Siliski (1993) [28]	26	7	0	18	0	1	0	0	0	0	0
Sneppen (1971) [29]	23	20	0	3	0	0	0	0	0	0	0
Sneppen (1971) [30]	6	0	0	2	2	0	1	1	0	33% arthrosis	0
Walsh (2004) [12]	6	1	0	5	0	0	0	0	0	1 persistent pain	0
Total	183 (100%)	63 (34.4%)	6 (3.2%)	97 (53%)	6 (3.2%)	2 (1.0%)	1 (0.5%)	1 (0.5%)	7 (3.8%)	7 (3.8%)	

**Table 4:** Type of ORIF (open reduction internal fixation).

Type of ORIF	97 (100%)
Plate	77 (79.4%)
Intramedullary Nail	4 (4.1%)
Percutaneous screw	12 (12.4%)
Lag screw	4 (4.1%)

## Data Availability

The data are available on request to the corresponding author.

## Supplementary Materials

The following supporting information can be downloaded at: https://www.mdpi.com/article/10.3390/jpm16070373/s1, Table S1: Missing Data from the included studies.

## Abbreviations

The following abbreviations are used in this manuscript:

BG	Bone Graft
CT	Computed tomography
EF	External Fixator
FX	Fracture
HTO	High tibial osteotomy
N/A	Not available
ORIF	Open reduction and internal fixation
PRISMA	Preferred reporting items for systematic reviews and meta-analyses
