# Supplementary material for: Fibular Nonunion: A Systematic Review of Incidence, Diagnosis, and Treatment Outcomes
Source: J Pers Med. 2026 Jul 10;16(7):373. doi: 10.3390/jpm16070373 (PMC13412602; doi:10.3390/jpm16070373)
Supplement: Supplementary file 1 [file jpm-16-00373-s001.zip › jpm-4305505-supplementary.pdf]

Variable	Available patients	Missing patients
**Age**	120	63 (34.4%)
**Sex**	113	70 (38.2%)
**Diagnostic Assessment**	183	0 (0%)
**Time to diagnosis**	95	88 (48.1%)
**Site of fibular nonunion**	168	15 (8.2%)
**Symptoms**	183	0 (0%)
**Previous treatment**	103	80 (43.7%)

Supplementary Table S1. Missing Data from the included studies.
